# Development of a light-regulated cell-recovery system for non-photosynthetic bacteria

**DOI:** 10.1186/s12934-016-0426-6

**Published:** 2016-02-15

**Authors:** Mitsuharu Nakajima, Koichi Abe, Stefano Ferri, Koji Sode

**Affiliations:** Department of Biotechnology and Life Science, Graduate School of Engineering, Tokyo University of Agriculture & Technology, 2-24-16 Naka-cho, Koganei, Tokyo 184-8588 Japan; Japan Science and Technology Agency, CREST, 2-24-16 Naka-cho, Koganei, Tokyo 184-8588 Japan; Department of Applied Chemistry and Biochemical Engineering, Shizuoka University, 3-5-1 Johoku, Naka-ku, Hamamatsu, Shizuoka 432-8561 Japan

**Keywords:** Antigen 43, Green light induction, Cell recovery, Green light sensor, Optogenetics, *Escherichia coli*

## Abstract

**Background:**

Recent advances in the understanding of photosensing in biological systems have enabled the use of photoreceptors as novel genetic tools. Exploiting various photoreceptors that cyanobacteria possess, a green light-inducible gene expression system was previously developed for the regulation of gene expression in cyanobacteria. However, the applications of cyanobacterial photoreceptors are not limited to these bacteria but are also available for non-photosynthetic microorganisms by the coexpression of a cyanobacterial chromophore with a cyanobacteria-derived photosensing system. An *Escherichia coli*-derived self-aggregation system based on Antigen 43 (Ag43) has been shown to induce cell self-aggregation of various bacteria by exogenous introduction of the Ag43 gene.

**Results:**

An *E. coli* transformant harboring a plasmid encoding the Ag43 structural gene under a green light-regulated gene expression system derived from the cyanobacterium *Synechocystis* sp. PCC6803 was constructed. Ag43 was inserted downstream of the *cpcG*_*2*_ promoter P_*cpcG2*_, and its expression was regulated by green light induction, which was achieved by the functional expression of cyanobacterial CcaS/CcaR by coexpressing its chromophore synthesis gene cassette in *E. coli*. *E. coli* transformants harboring this designed system self-aggregated under green light exposure and precipitated, whereas transformants lacking the green light induction system did not. The green light induction system effectively functioned before the cell culture entered the stationary growth phase, and approximately 80 % of the cell culture was recovered by simple decantation.

**Conclusion:**

This study demonstrated the construction of a cell recovery system for non-photosynthetic microorganisms induced by exposure of cells to green light. The system was regulated by a two-component regulatory system from cyanobacteria, and cell precipitation was mediated by an autotransporter protein, Ag43. Although further strict control and an increase of cell recovery efficiency are necessary, the system represents a novel tool for future bioprocessing with reduced energy and labor required for cell recovery.

## Background

Recent advances in the understanding of photosensing in biological systems have permitted the use of photoreceptors as novel genetic tools [1–8]. Photoreceptors are protein machineries that detect and respond to changes in light quality and intensity. Optogenetics, which uses various photoreceptors to control cell behaviors directly via light exposure, has recently attracted attention as a synthetic biology-based bioprocess design.

Cyanobacteria have various light-sensing systems to effectively regulate photosynthesis [9, 10] and avoid photo-inhibition caused by strong or short-wavelength light [11–17]. By exploiting various photoreceptors in cyanobacteria, a green light-inducible gene expression system has been developed. A unicellular cyanobacterium, *Synechocystis* sp. PCC6803, harbors a green light-sensing system. The expression of a phycobilisome linker gene, *cpcG2*, is chromatically regulated by a sensor histidine kinase, CcaS, and a cognate response regulator, CcaR [18]. Using the endogenous CcaS/CcaR system, the green light regulation of an exogenously induced gene was achieved using a modified promoter of *cpcG2*, P_*cpcG2*_, inserted upstream of the target gene on a vector plasmid [19]. In addition, CcaS, CcaR, and P_*cpcG2*_ from *Synechocystis* sp. PCC6803 has been transformed into the marine cyanobacterium *Synechococcus* sp. NKBG 15041c as an exogenous green light-regulated gene expression system [20]. This system has been applied to the construction of a green light-regulated autolysis system for cyanobacteria that employs a T4 phage-derived lysis system under the control of green light-regulated gene expression [21].

However, the applications of cyanobacterial photoreceptors are not limited to cyanobacteria but are also available for non-photosynthetic microorganisms. A pioneering study by Tabor et al. achieved the functional expression and utilization of a cyanobacterium-derived green light-sensing system in *E. coli* [22]. Because phycocyanobilin (PCB), a chromophore of CcaS, is not endogenously synthesized in *E. coli*, the coexpression of a PCB synthesis gene cassette together with CcaS/CcaR resulted in green light-regulated gene expression in *E. coli* [23]. Tabor and his coworkers also reported a multichromatic gene expression system employing an engineered CcaS.

In this study, we aimed to construct a novel technology for non-photosynthetic microorganism-based bioprocesses, a light-regulated cell-recovery system. As a light-regulated gene expression system, the cyanobacterium-derived green light-regulated gene expression system controlled by the two-component regulatory system CcaS/CcaR was selected. For the cell recovery technology, the *E. coli*-derived self-aggregation system was selected. Antigen 43 (Ag43), an autotransporter protein from *E. coli*, is an essential protein for aggregation and biofilm formation during infection. Ag43 is composed of three domains: a signal peptide for secretion into the periplasmic space, a β domain that forms a selective channel in the outer membrane to transfer the α domain for extracellular display, and an α domain, which is a linker for the self-aggregation. High affinity among the α domains triggers self-aggregation, which leads to cell precipitation [24–28]. Recently, the structure of the α-domain complex of Ag43 has been reported [29]. In the present study, the Ag43 structural gene was inserted downstream of the *cpcG*_*2*_ promoter, P_*cpcG2*_, and its expression was regulated by green light induction, achieved by the functional expression of cyanobacterial CcaS/CcaR by coexpression of its chromophore synthesis gene cassette in *E. coli*. *E. coli* transformants carrying this system self-aggregated under green light exposure and precipitated, whereas transformants lacking the green light-induction system did not. The green light-induction system effectively functioned before the cell culture entered the stationary growth phase, and approximately 80 % of the cell culture was recovered by simple decantation.

## Methods

### Construction of a plasmid encoding a green light-inducible aggregation system

**Fig. 1:**
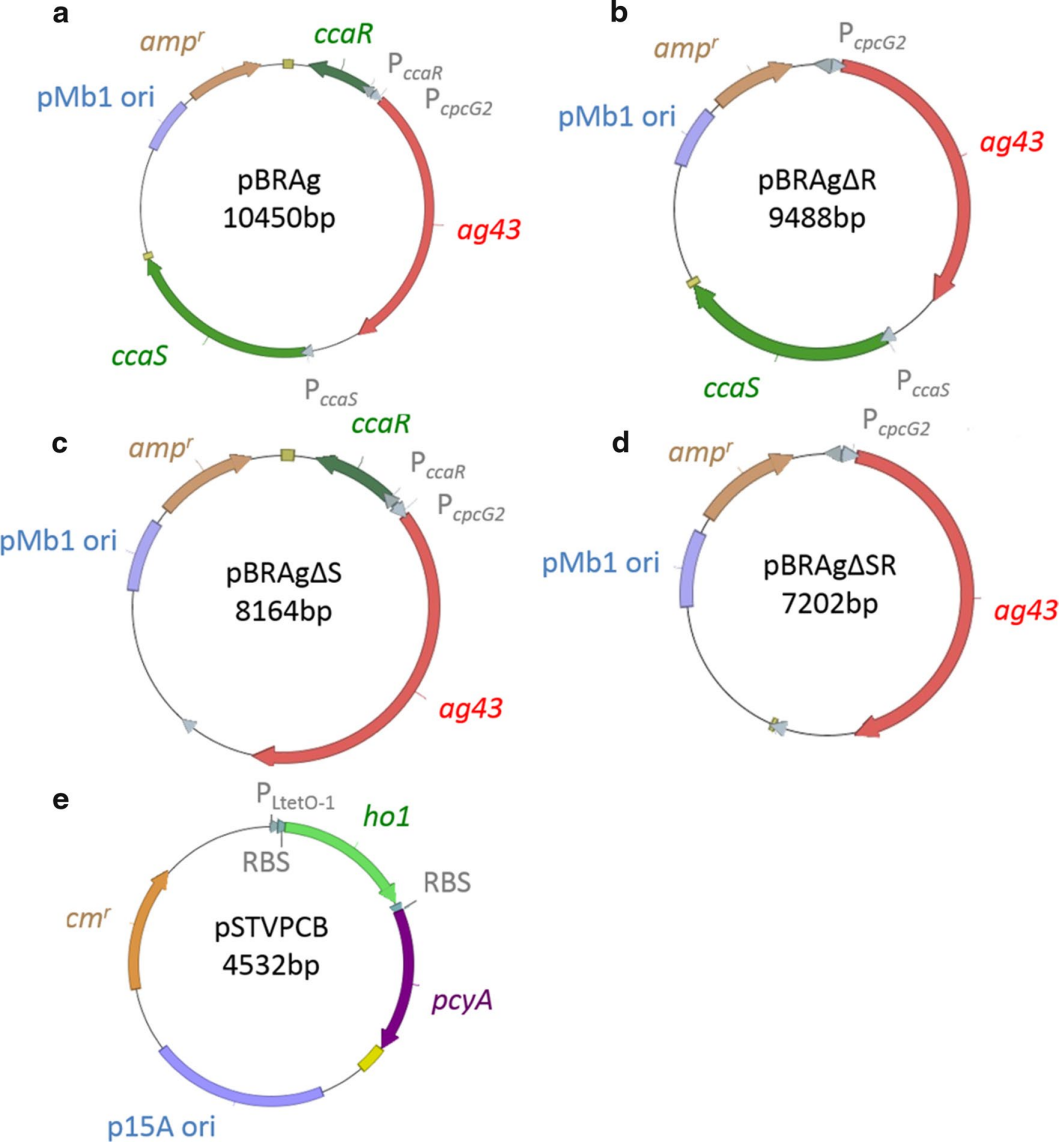
Plasmid vectors used in this study. Plasmid shown in (**a**) encodes a gene necessary for aggregation (*ag43*) and an entire green light-sensing system (**a** pBRGLAg). Plasmids shown in (**b**), (**c**) encode green light-sensing system but lacking gene encoding CcaR (**b** pBRGLAgΔR, or gene encoding CcaS (**c** pBRAgΔS). Plasmid shown in (**d**) encodes a gene necessary for aggregation (*ag43*) but not the genes for green light-sensing system (**d** pBRGLAgΔSR). The plasmid shown in (**e**) encodes phycocyanobilin (*PCB*) synthetic genes, pSTVPCB

Gene encoding the aggregation protein, *ag43* derived from *E. coli* amplified from BioBrick BBa_K317008 (Registry of Standard Biological Parts [30]) was inserted downstream of a P_*cpcG2*_ promoter corresponding to the *Nde*I and *Xba*I sites of pKTGSS, which contains the green light-sensing two-component regulatory system CcaS/CcaR [20]. The resulting plasmid was designated as pKTGLAg. The gene cassette containing the green light-inducible aggregation system was amplified by PCR using primers 5′-AGCGGCCGCGAATTCTTGAAGACGAAAGGGCCTC-3′ and 5′-TTTTTTCGCCTGCAGATGGAAGCCGGCGGCAC-3′ and pKTGLAg as a template. The plasmid pBR322 was linearized and the region, except for the TetC-coding gene, was amplified using primers 5′-GAATTCGCGGCCGCTTCTAG-3′ and 5′- CTGCAGGCGAAAAAACCCCGCCGAAG-3′ [31]. These amplified products were fused with an In-Fusion cloning kit (Takara, Otsu, Japan). The constructed vectors comprising the green light-inducible aggregation system (pBRGLAg) are shown in Fig. 1. Control vectors with each component deleted were also constructed by inverse PCR using pBRGLAg with primers designed to eliminate *ccaS* or *ccaR* (pBRGLAg∆S, pBRGLAg∆R, and pBRGLAg∆SR). These constructed vectors are shown in Fig. 1, and the components are described in Table 1.

**Table 1:** Vector used in this study

Plasmid name	Origin	Resistance	Feature	Source
pKTGSS	V ori, p15A ori	Streptomycin	*cca* cluster with *gfpuv* instead of *cpcG2*	Ref. [20]
pBRGLAg	pMb1 ori	Ampicillin	*cca* cluster with *ag43* instead of *cpcG2*	This study
pBRGLAg∆S	pMb1 ori	Ampicillin	*cca* cluster with *ag43* instead of *cpcG2* without *ccaS*	This study
pBRGLAg∆R	pMb1 ori	Ampicillin	*cca* cluster with *ag43* instead of *cpcG2* without *ccaR*	This study
pBRGLAg∆SR	pMb1 ori	Ampicillin	*cca* cluster with *ag43* instead of *cpcG2* without *ccaS*, *ccaR*	This study
pBR322	pMb1 ori	Ampicillin/tetracycline	original vector used for construction of pBRGLAg	Ref. [31]
pSTVPCB	p15A ori	Chloramphenicol	*ho1* and *pcyA* genes for PCB synthesis	This study
pSTV28	p15A ori	Chloramphenicol	original vector used for construction of pSTV28	Ref. [33]

### Construction of a plasmid encoding PCB synthesis genes

A PCB synthesis gene cassette was constructed by assembling P_*LtetO*-*1*_ (BBa_R0040; Registry of Standard Biological Parts [30]), a ribosomal binding site (RBS) (BBa_B0034; Registry of Standard Biological Parts [30]), the heme oxygenase gene *ho1* from *Synechocystis* sp. PCC6803 (BBa_I15008; Registry of Standard Biological Parts [30]), the PCB–thioredoxin oxidoreductase gene *pcyA* from *Synechocystis* sp. PCC6803 (BBa_I15009; Registry of Standard Biological Parts [30]), and a double terminator (BBa_B0015; Registry of Standard Biological Parts [30]) using three antibiotic assembly and inserted at the *Eco*RI and *Pst*I sites of the plasmid derived from pSTV28, whose construction has been previously described [32, 33, 34]. This plasmid was named pSTVPCB. In this plasmid, *ho1* and *pcyA* were constitutively transcribed independently following RBS in *E. coli* DH5α by P_*Ltete*-*1*_ polycistronically (Fig. 1). The components of this plasmid are shown in Table 1.

### Autoaggregation-regulation assay

*E. coli* cells carrying pBRGLAg, pBRGLAgΔS, pBRGLAgΔR, or pBRGLAgΔSR together with pSTVPCB were cultured in LB broth containing 25 µg/ml chloramphenicol and 100 µg/ml ampicillin in a test tube at 37 °C with shaking at 140 rpm overnight. The prepared pre-cultures were inoculated into fresh 40 ml LB broth containing 0.1 M HEPES (pH 6.6), 0.05 mM aminolevulinic acid, 0.05 mM FeCl_3_, 100 µg/ml ampicillin, and 25 µg/ml chloramphenicol in 100-ml Erlenmeyer flasks. Cell density was monitored 6 h after the start of culture. Cells were cultured with shaking at 100 rpm and exposed to red light (660 nm, 40 µmol s^−1^ m^2^) at 30 °C until the cell density reached OD_595_ or OD_600_ = 0.4–0.6. After this period, each transformant was cultured under either of the following two conditions: one culture in triplicate was exposed to green light (520 nm, 40 µmol s^−1^ m^2^) instead of red light for 6 h, and the other culture in triplicate was continuously exposed to red light with shaking at 100 rpm and 30 °C. A 10-ml culture was transferred to a 15-ml tube to measure the aggregation-regulation ability of the cells.

The transferred culture in each 15-ml tube was exposed to red light for 2 h. During the incubation, a 100-µl culture was periodically transferred from the tube to a 96-well plate every 10 min, and 200 µl of fresh culture was added to the wells to dilute the culture. Cell density was measured using a plate reader (Thermo Fisher Scientific Inc., MA, USA). Cell density measurements were performed in triplicate. In all aggregation experiments, *E. coli* DH5α was used.

### Transcriptional analysis of *ag43* by quantitative reverse transcription PCR

*E. coli* cells harboring pSTVPCB and pBRGLAg were cultured as described above in the aggregation-regulation assay. During culture, a 1-ml culture was periodically removed.

Total RNA was extracted from the cell pellets from 1-ml cultures taken after centrifugation at 12,000 g for 5 min at 4 °C, using a NucleoSpin^®^ RNA Clean-up kit (Takara Bio Inc., Shiga, Japan). The extracted RNA was treated with DNase to eliminate genomic DNA, and reverse transcription from RNA to cDNA was performed using PrimeScript^®^ RT reagent kit with gDNA Eraser (Takara Bio Inc.). Quantitative PCR was performed to measure the transcriptional level of *ag43* and 16S ribosomal RNA (rRNA) (housekeeping genes) with SYBR^®^ Premix Ex TaqTM II (Tli RNaseH Plus) (Takara Bio Inc.). The transcription level was measured using the ΔΔCt method and normalized using the calculated transcription values of 16S rRNA.

### Evaluation of cell recovery

Cells harboring the green light-inducible aggregation system were cultured as described above with modification in the timing of the start of exposure to green light.

To determine the timing of gene induction, cultures were induced by green light at different stages of growth. Four separate cultures in triplicate were prepared. For each culture, green light was irradiated at OD_595_ = 0.7, 1.1, or 1.2 or until 10 h had passed after the cell density reached OD_595_ = 1.7. Cultures were then exposed to green light (520 nm, 40 µmol s^−1^ m^2^) for 2 h.

Cultures diluted to cell density OD_595_ = 1.0 by the addition of fresh LB broth containing 0.1 M HEPES (pH 6.6), 0.05 mM aminolevulinic acid, 0.05 mM FeCl_3_, 100 µg/ml ampicillin, and 25 µg/ml chloramphenicol were transferred to a 15-ml tube and exposed to red light for 180 min for cell precipitation. Then, 7.6 ml of the supernatant was sampled and 400 µl of the culture containing precipitated cells was left behind (decantation procedure). The remained cells in 400 µl of the culture was defined as the recovered cells. In order to quantify the amount of recovered cells and unrecovered cells, thus prepared 400 µl of the culture containing precipitated cells and 7.6 ml supernatant were centrifuged. The cell recovery was calculated as the ratio (%) of the wet weight cells of recovered cells and total (recovered and unrecovered) cells.

## Results

### Light-regulated precipitation

**Fig. 2:**
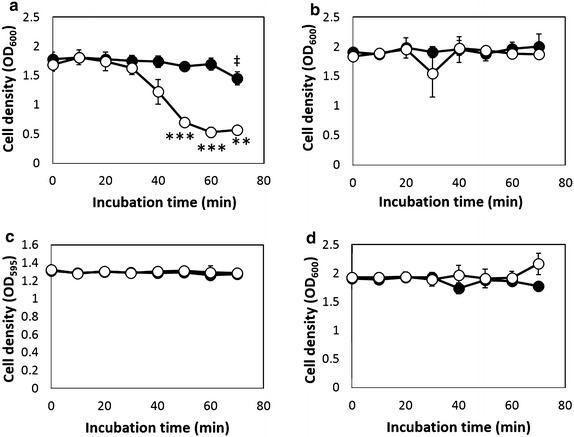
Time course of aggregation of cells harboring a green light-inducible aggregation system. *White circles* show the time course of aggregation of cells grown under *green light* and *black circles* show the time course of aggregation of cells grown under *red light*. OD_600_ or OD_595_ were used as cell density indicators. **a** Cells harboring a green light-inducible aggregation system; **b** cells harboring a green light-inducible aggregation system lacking CcaS, the green light-sensor protein; **c** cells harboring a green light-inducible aggregation system lacking CcaR, the cognate response regulator of CcaS; **d** cells harboring a green light-inducible aggregation system lacking CcaS and CcaR. The experiment was performed in triplicate. The double dagger indicates statistical difference in the cell density under *red light* from 0 min incubation (‡P < 0.05). The *asterisks* indicate statistical differences from the cells exposed to *red light* (**P < 0.01 and ***P < 0.001 based on Dunnet’s t test)

*E. coli* transformants were grown under red light (660 nm, 40 µmol m^−2^ s^−1^) until the cell density reached the exponential growth phase (OD_595_ or OD_600_ = 0.5–0.6). Red light exposure was then ceased, and the culture was continued under green light exposure. After 6 h of green light exposure, the precipitation of the cells was measured. Figure 2a–d show the time courses of cell precipitation, including those harboring pSTVPCB for PCB synthesis and pBRGLAg encoding CcaS/CcaR and Ag43 under P_*cpcG2*_ (2a), pSTVPCB and pBRGLAg∆S (without CcaS) (2b), pSTVPCB and pBRGLAg∆R (without CcaR) (2c), or pSTVPCB and pBRGLAg∆SR (without CcaS/CcaR) (2d). The transformants harboring pSTVPCB and pBRGLAg exposed to green light (520 nm, 40 µmol m^−2^ s^−1^) started to decrease in OD_600_ during incubation, indicating that cell precipitation had started. After 60 min of incubation, the decrease in OD_600_ ceased at approximately OD_600_ = 0.5, indicating that cell precipitation had stopped. At this point, >50 % of the cells had precipitated. However, no precipitation was observed for transformants harboring pSTVPCB and pBRGLAg, which were exposed only to red light prior to the precipitation assay. In contrast, transformants harboring pSTVPCB and pBRGLAg∆S, pBRGLAg∆R, or pBRGLAg∆SR, cultured under red or green light, showed no decrease in OD_595_ or OD_600_, suggesting that these transformants had no precipitation ability (Fig. 2b, c, d). These results indicated that cell precipitation ability was induced when the transformants harbored both genes, those for PCB synthesis and for the complete two-component regulatory system CcaS/CcaR, and only when cultured under green light.

**Fig. 3:**
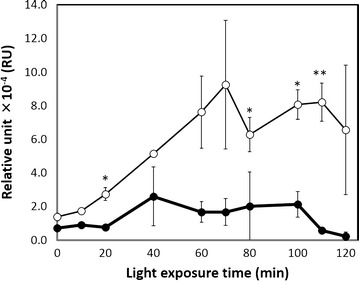
Transcriptional analyses of Ag43 under *green light* induction. Relative units of *ag43* transcription were normalized to 16S rRNA amounts. Relative units in cells grown under *green light* are shown with *white circles*, and relative units in cells grown under *red light* are shown with *black circles*. There is no significant difference in the cell density under *red light* at each incubation time from at 0 min incubation. The *asterisks* indicate statistical difference from the cells exposed to *red light* (*P < 0.05 and **P < 0.01, based on Dunnet’s t-test)

Transcriptional analysis of *ag43* in transformants harboring pSTVPCB and pBRGLAg was performed. The transformants were cultured under red light for 12 h and then cultured under green light for the next 2 h, whereas the other transformants were cultured under red light for 14 h. Transcriptional analysis was performed for cells cultured after the first 12 h of culture (Fig. 3). The transcriptional level of *ag43* gradually increased after exposure to green light for 20 min. The transcriptional level reached to the highest level by 80 min incubation and remained stable for at least the next 20 min at least. Although an increase tendency of *ag43* transcription was observed when the culture was exposed to red light, the transcriptional level was <30 % of that observed 80–110 min after exposure to green light.

The results of the aggregation assays and transcriptional analysis indicated that the green light regulated aggregation of *E. coli* cells was achieved by introducing a green light-sensing two-component regulatory system derived from cyanobacteria and Ag43 gene.

### Cell recovery

**Fig. 4:**
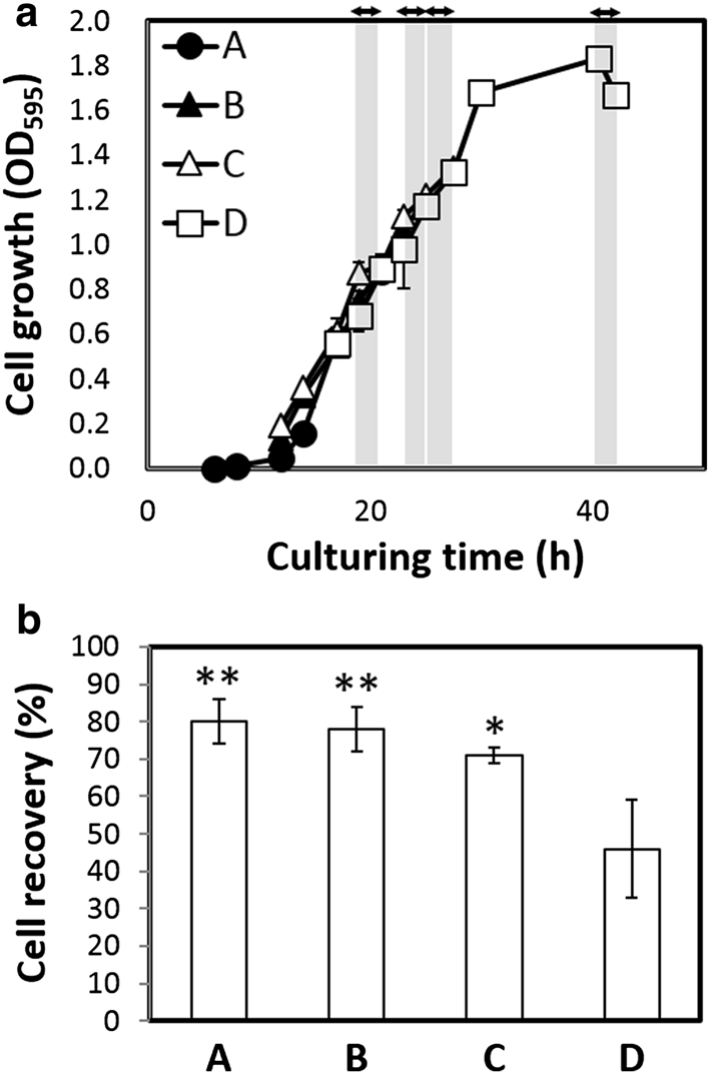
Evaluation of cell recovery under *green light* induction at various cell growth phases. **a** Time course of cell growth. **b** Cell recovery from cultures exposed to *green light* for 2 h. Cell recovery is shown as the ratio (%) of recovered cells (wet weight) to total cells (wet weight) in culture in a tube. The *asterisks* indicate statistical differences from the cells exposed to *green light* on stationary phase (*P < 0.05 and **P < 0.01 based on Dunnet’s t-test). *A* Cells exposed to *green light* at 19–21 h of culture, early log phase; *B* cells exposed to *green light* at 23–25 h of culture, middle log phase. *C* Cells exposed to *green light* at 25–27 h of culture, late log phase; *D* cells exposed to *green light* at 40–42 h of culture, stationary phase

The engineered *E. coli* cells harboring pSTVPCB and pBRGLAg were then subjected to investigation of cell recovery. Cells were cultured under green or red light (Fig. 4). After an approximately 12-h lag phase, cell growth entered the logarithmic growth phase and reached the stationary phase after 30 h. Cells at each growth phase, the early logarithmic growth phase (12–18 h; OD_595_ = approximately 0.7), mid-logarithmic growth phase (18–28 h; OD_595_ = approximately 1.0), late logarithmic growth phase (28–32 h; OD_595_ = approximately 1.8), and stationary phase (10 h after the late log phase), were exposed to green light to induce precipitation. Cultures containing cells exposed to green light at each growth phase were diluted to OD_595_ = 1.0 by addition of LB broth, and an 8 ml culture of each was transferred to a 15-ml tube. Tubes were exposed to red light.

Cells at all growth phases showed precipitation ability, with 2-h exposure to green light resulting in Ag43 expression. Amounts of cells recovered by decantation of the precipitated cells are shown in Fig. 4. From the cells exposed to green light at the early, middle, and late-logarithmic growth phases, >70 % of cells were recovered by decantation. However, for cells exposed to green light at the stationary phase, <50 % of total number of cells was recovered by decantation. Thus, for efficient recovery of engineered *E. coli* cells harboring pSTVPCB and pBRGLAg, cells should be exposed to green light before growth enters the stationary phase, preferably before the late logarithmic phase. These results demonstrate the construction of a green light-induced cell recovery system for non-photosynthetic microorganisms by the combination of a cyanobacteria-derived green light-sensing system and Ag43 from *E. coli*.

## Discussion

In this study, we aimed to construct a green light-regulated cell recovery system for non-photosynthetic microorganisms using a green light-regulated gene expression system controlled by a two-component regulatory system from cyanobacteria and using Ag43, an autotransporter protein from *E. coli*.

Recently, the crystal structure of α-domain of Ag43 has been reported [29]. The crystal structure of this domain shows that the formation of cell aggregates proceeds via a molecular Velcro-like handshake mechanism. Under this mechanism, if Ag43 is expressed on the surface of the outer membrane, cell self-aggregation will occur. The self-aggregation of bacteria using recombinant Ag43 has been previously reported [28]. Exogenously introduced Ag43 led to the self-aggregation of *E. coli*, *Pseudomonas fluorescens*, and *Klebsiella pneumoniae*. Thus, our green light-induced cell recovery system will also be useful in a variety of non-photosynthetic microorganisms if the functional expression of the green light-sensing system is possible with the introduction of the PCB synthesis gene cassette.

Cell precipitation was observed in green light-exposed transformants harboring both pSTVPCB for PCB synthesis and pBRGLAg encoding CcaS/CcaR and Ag43 under P_*cpcG2*_ but not in transformants harboring pSTVPCB and with an imperfect green light-regulation system (pBRGLAg∆S, pBRGLAg∆R, or pBRGLAg∆SR) (Fig. 2a–d). However, even in the transformants with pSTVPCB and pBRGLAg, slight precipitation was observed under red light exposure at 70 min of incubation (Fig. 2a). Although the slight precipitation of the cells exposed to red light was observed at 70 min incubation, the expression of Ag43 under red light was not observed by transcriptional analysis (Figs. 2, 3). However, because the increase tendency was observed in the *ag43* transcription under red light, the result suggests undetectable level *ag43* transcription under red light led to Ag43 expression and precipitation of cells under red light. The difference in the Ag43 expression levels of transformants with pSTVPCB and pBRGLAg under red light was obvious in transformants harboring an imperfect green light-sensing system with *ag43*. Thus, the expression of Ag43 under red light was not due to the endogenously present P_*cpcG2*_ activating factors in *E. coli* but due to background-level expression under red light in the presence of CcaS/CcaR. It has been reported that CcaS autophosphorylation was repressed under red light. To prevent expression leakage of Ag43 under non-inducing conditions, we cultured *E. coli* transformants under red light. However, further repression of kinase activity of CcaS is required to achieve tight regulation using this system.

Cell recovery by exposure to green light was achieved when the cells were induced before entry into the stationary phase. However, when cells were exposed to green light even at the early-logarithmic growth phase, 80 % could be recovered by decantation with 20 % remaining in the culture supernatant. Aggregation is strongly dependent on the cell concentration [27]. With decreasing free cell concentration in the supernatant resulting from the precipitation of flocculate from Ag43-mediated aggregation, aggregation may decrease. To overcome this inherent problem of aggregation-mediated cell recovery, an increase in the expression level of Ag43 per cell would enhance cell precipitation.

## Conclusions

In conclusion, this study demonstrated the construction of a cell recovery system for non-photosynthetic microorganisms that is induced by the exposure of cells to green light. The system is regulated by a two-component regulatory system from cyanobacteria, and the cell precipitation is mediated by an autotransporter protein, Ag43. Although further strict control and increase of cell recovery efficiency are necessary, the proposed system provides a novel tool for future bioprocessing with reduced energy and labor for cell recovery.
